# Systems-Level Transcriptomic Integration Reveals a Core Metaflammatory Network Linking Type 2 Diabetes and HBV Infection to Cholangiocarcinoma Progression

**DOI:** 10.3390/cancers18060923

**Published:** 2026-03-12

**Authors:** Hasan Md Rasadul, Shihui Ma, Ziqiang Ge, Rahman Md Zahidur, Pengcheng Kang, Junqi You, Jinglin Li, Chenghong Duan, Siddique A. Z. M. Fahim, Mozumder Somrat Akbor, Xudong Zhao, Yunfu Cui

**Affiliations:** 1 Department of Biliary-Pancreatical Surgery, The Second Affiliated Hospital of Harbin Medical University, Harbin 150086, China; mdrasel3051106@gmail.com (H.M.R.); mashihui@hrbmu.edu.cn (S.M.); geziqiangg@163.com (Z.G.); kang_pengcheng@126.com (P.K.); youjunqi18@163.com (J.Y.); lijinglin77@hrbmu.edu.cn (J.L.); 2023021637@hrbmu.edu.cn (C.D.); xdzhao9@163.com (X.Z.); 2 North Care Consultation Hub, Rangpur 5405, Bangladesh; drzahid2003@gmail.com; 3 Internal Medicine, Department of Cardiology, The First Affiliated Hospital of Harbin Medical University, Harbin 150001, China; drfahimsiddique@gmail.com; 4 The Second Affiliated Hospital of Harbin Medical University, Harbin 150086, China; somratpaponsp@gmail.com

**Keywords:** cholangiocarcinoma (CCA), type 2 diabetes mellitus (T2D), hepatitis B virus (HBV), metaflammation, transcriptomics, systems biology, prognostic biomarker, drug repurposing

## Abstract

Background and Aims: The rising global incidence of cholangiocarcinoma (CCA) coincides with epidemics of type 2 diabetes (T2D) and chronic hepatitis B virus (HBV) infection. Although both are established independent risk factors, the shared molecular mechanisms by which they contribute to cholangiocarcinogenesis remain poorly understood. We hypothesized that T2D and HBV converge on a state of chronic metabolic inflammation (“metaflammation”) that drives CCA progression through a conserved transcriptomic network. Methods: We performed an integrative bioinformatics analysis of transcriptomic data from public repositories, including samples of CCA (TCGA-CHOL, *n* = 45; GSE107943, *n* = 163), T2D-affected liver (GSE23343, *n* = 20), and HBV-infected liver (GSE58208, *n* = 102). Acknowledging that the T2D and HBV datasets were derived from whole-liver tissue, whereas CCA originates in the biliary epithelium, we identified differentially expressed genes (DEGs) across conditions and defined a core gene set shared among them. Subsequent analyses included functional enrichment, construction of protein–protein interaction (PPI) networks, survival analysis, and protein validation. Results: We identified a core metaflammation signature comprising 156 genes that were consistently dysregulated across T2D, HBV, and CCA. Pathway analysis revealed significant enrichment in PPAR signaling, cytokine–cytokine receptor interaction, PI3K-Akt, and TNF signaling pathways. Protein–protein interaction (PPI) network analysis identified IL6, TNF, AKT1, STAT3, and PPARG as the top hub genes. These hubs were functionally modularized into clusters associated with inflammatory signaling, metabolic regulation, and cell growth and survival. In the TCGA CCA cohort, high expression of IL6, TNF, AKT1, and STAT3 and low expression of PPARG correlated with advanced tumor stage and poorer overall survival (e.g., IL6: ρ = 0.42, *p* = 0.01). A metaflammation score derived from these hubs (weighted combination of the five genes) emerged as an independent prognostic factor (HR = 2.8, *p* < 0.001). Protein-level dysregulation of these hubs was confirmed via immunohistochemistry. Conclusions: This study defines a conserved metaflammation network that links T2D and HBV to CCA, identifying key hub genes and pathways. This signature provides a mechanistic explanation for epidemiological risks, serves as a novel prognostic tool, and offers a rationale for targeting metaflammation in prevention and therapy for high-risk populations.

## Simple Summary

Cholangiocarcinoma, a malignancy of the bile ducts, is associated with poor survival, and its incidence is rising globally. This trend parallels the rising epidemics of type 2 diabetes mellitus and chronic hepatitis B infection. Although these conditions are recognized risk factors for cancer, the underlying biological mechanisms remain poorly understood. In this study, we conducted an integrative analysis of genetic data from patients with these three diseases to identify potential molecular links. Our analysis revealed a shared set of 156 genes, implicating a state of chronic inflammation driven by metabolic dysregulation that connects diabetes and hepatitis B infection to cholangiocarcinogenesis. Within this network, five key genes were significantly associated with patient survival. These findings provide a molecular framework that elucidates how these risk factors contribute to cancer development. This research opens new avenues for identifying at-risk individuals and suggests that targeting this specific inflammatory pathway may offer novel strategies for cancer prevention and treatment.

## 1. Background

Cholangiocarcinoma (CCA), a malignancy arising from the biliary tract epithelium, remains a formidable clinical challenge. It is characterized by late diagnosis, therapeutic resistance, and a dismal 5-year survival rate, often below 20% [1], underscoring a critical unmet need in oncology. The etiological landscape of CCA is complex and evolving. Although primary sclerosing cholangitis and liver fluke infections are well-established risk factors, a modern epidemiological shift implicates systemic metabolic dysfunction and chronic viral hepatitis as major drivers of rising incidence, particularly in Western populations [2,3].

Two interconnected global health burdens, type 2 diabetes (T2D) and chronic hepatitis B virus (HBV) infection, have emerged as significant independent risk factors for CCA. Meta-analyses indicate that T2D confers a 1.5- to 2.0-fold increased risk of CCA, particularly the intrahepatic subtype (iCCA), with risk correlating with disease duration and glycemic severity [4,5]. Concurrently, HBV, a canonical cause of hepatocellular carcinoma, is now robustly associated with an elevated risk of CCA, with viral components detected within cholangiocytes [6].

These conditions converge pathophysiologically within the liver, fostering a state of “metaflammation” defined as persistent, low-grade inflammation driven by metabolic dysfunction. T2D contributes to hyperinsulinemia, lipotoxicity, and adipokine imbalance, whereas HBV drives immune-mediated injury and direct viral oncoprotein signaling (e.g., HBx) [7]. Collectively, these factors create a permissive microenvironment characterized by oxidative stress, altered growth factor signaling, and immune dysregulation, thereby promoting genomic instability and uncontrolled proliferation.

Despite compelling epidemiological evidence linking T2D and HBV to CCA, the precise and conserved molecular mechanisms underlying this association remain inadequately defined. A critical gap persists in our understanding of the shared transcriptomic architecture and functionally interconnected pathways dysregulated across this disease triad. Single-cohort studies lack the statistical power to distinguish universal drivers from biological noise [8]. Therefore, a systems-level integrative analysis of multi-condition transcriptomic data is essential to decode the shared pathogenic network.

Leveraging high-throughput genomic data from large public repositories (TCGA and GEO), this study employs a comprehensive bioinformatics framework to test the central hypothesis that T2D and chronic HBV infection promote cholangiocarcinogenesis through a core set of dysregulated metabolic-inflammatory driver genes embedded within central regulatory networks. Our specific objectives are to: (1) define condition-specific and shared transcriptomic alterations; (2) elucidate the functional architecture and network structure of the shared gene set; (3) determine the clinical and prognostic significance of this metaflammation signature; and (4) validate key findings at the protein level. This integrative approach seeks to move beyond association toward mechanistic elucidation, with the goal of identifying novel biomarkers and therapeutic targets for CCA prevention and treatment in globally significant at-risk populations.

## 2. Materials and Methods

### 2.1. Study Design and Data Acquisition

**Figure 1:**
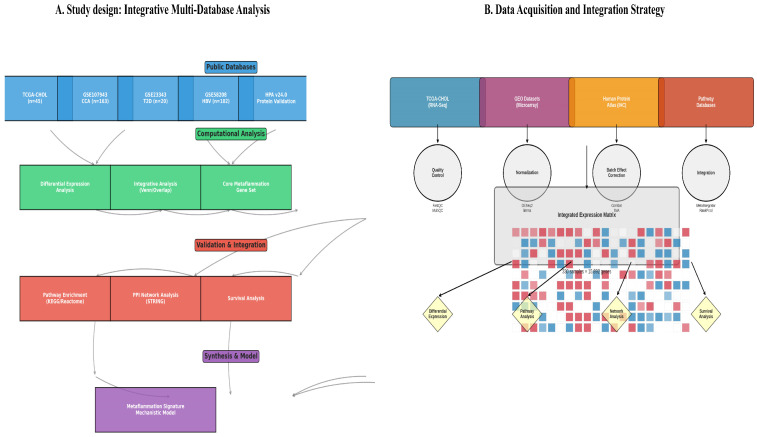
(**A**) Study design for the identification and validation of a metaflammation signature. The workflow involved differential expression analysis of several disease-specific datasets: TCGA-CHOL (cholangiocarcinoma, *n* = 45), GSE107943 (CCA, *n* = 163), GSE23343 (Type 2 Diabetes, *n* = 20), and GSE58208 (HBV infection, *n* = 102). A Venn analysis identified a consistent core set of genes altered across these conditions. This core gene set was validated using data from the Human Protein Atlas and subsequently analyzed using pathway enrichment (KEGG/Reactome), protein–protein interaction networks (STRING), and clinical survival analysis. The findings were synthesized to define a metaflammation signature and construct a model linking chronic metabolic inflammation to disease pathogenesis. (**B**) Workflow for the integrative transcriptomic analysis and validation. The schematic describes a bioinformatics pipeline beginning with transcriptomic data acquisition from the TCGA-CHOL cohort and GEO (GSE107943, GSE23343, GSE58208) microarray datasets. Following preprocessing and normalization, differential expression analysis was conducted for conditions CCA, T2D, and HBV. The resulting gene lists were merged to identify a core metaflammation gene set, which was then subjected to functional enrichment and protein–protein interaction network analysis. The clinical relevance of key hub genes was evaluated in the TCGA cohort through survival analysis, and the protein-level expression of prioritized hubs was confirmed with immunohistochemistry data from the Human Protein Atlas. Note: CCA datasets are derived from bile duct tissue, while T2D and HBV datasets are from whole liver tissue. This tissue source heterogeneity is a key limitation discussed in the text.

This study employed an integrative bioinformatics approach combining multiple public databases to investigate molecular associations among T2D, HBV, and CCA; institutional review board (IRB) approval was not required. The analytical workflow encompassed data acquisition, preprocessing, differential expression analysis, integrative cross-condition analysis, functional enrichment, protein–protein interaction (PPI) network construction, survival analysis, and validation (Figure 1A).

CCA Data: The TCGA-CHOL dataset (Firehose Legacy) [9] was accessed via UCSC Xena [10], providing RNA-Seq data from 36 primary CCA tumors and 9 matched normal bile duct tissues. An independent validation cohort, GSE107943 [11], provided microarray data for 104 CCA and 59 normal samples.

Comorbidity Data: To model etiological risk factors, we utilized the following datasets: GSE23343 (microarray; 10 T2D vs. 10 control whole liver samples) [12] to derive a T2D metabolic signature, and GSE58208 (microarray; 62 HBV-positive vs. 40 HBV-negative whole liver samples) [13] to establish an HBV inflammatory signature. For exploratory contextual metabolic analysis, GSE89632 [12] (RNA-Seq; liver tissue across steatosis grades) was also employed. Although CCA originates from bile duct epithelium, these liver-derived datasets were selected as the most appropriate available proxies for the hepatic microenvironment that bathes and influences cholangiocytes in patients with metabolic and viral disease.

Validation Resources: The Human Protein Atlas (HPA v24.0) [14] provided immunohistochemistry (IHC) data for protein-level validation. Functional enrichment and network analyses were conducted using the KEGG, Reactome, STRING, and DisGeNET databases [15,16,17].

### 2.2. Data Preprocessing and Quality Control

To ensure comparability across platforms, platform-specific preprocessing pipelines were implemented. For RNA-Seq data (TCGA and GSE89632) [9,18], raw read counts were batch-corrected using ComBat-seq [19]. Differential expression analysis was performed directly on these counts using DESeq2 [20]. For visualization and signature scoring, counts were normalized using the trimmed mean of M-values (TMM) method [21] and subsequently variance-stabilized (VST). Microarray data (GSE107943, GSE23343, GSE58208) [11,12,13] were normalized using the Robust Multi-array Average (RMA) algorithm [18,22]. Datasets with available technical batch metadata (GSE107943 and GSE58208) were further corrected using ComBat [19]. For the GSE23343 (T2D) dataset [12], batch correction was not applied, as the available sample metadata did not indicate a processing batch structure, and preliminary principal component analysis (PCA) revealed no significant technical clustering.

**Figure 2:**
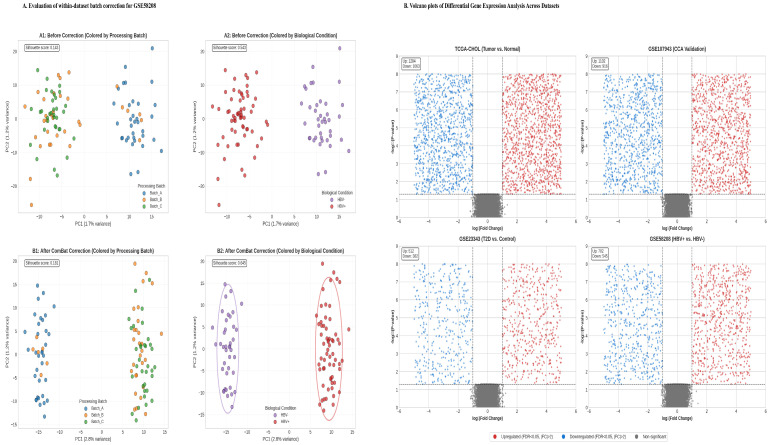
(**A**) Evaluation of within-dataset batch correction for microarray data. PCA plots of the GSE58208 (HBV) dataset show samples before and after batch correction, illustrating the effects of the ComBat algorithm. Initially, samples are colored by processing batch and biological condition (HBV+ vs. HBV-). After correction, the plots indicate reduced clustering by technical batch while maintaining clear separation by biological condition, demonstrating the effective removal of non-biological variance. Similar quality control assessments were conducted on other datasets, such as GSE107943. (**B**) Comparative Differential Gene Expression Analysis Across Datasets. Volcano plots show the results of differential gene expression across four transcriptomic studies comparing disease to control groups. The x-axis represents log_2_ fold change (log_2_FC) in gene expression, and the y-axis indicates statistical significance, marked by −log_10_(FDR). Dashed horizontal lines denote significance thresholds (FDR < 0.05), while vertical dashed lines indicate fold-change thresholds (|log_2_FC| > 1). Data points are highlighted in red for significantly upregulated genes (FDR < 0.05, log_2_FC > 1), blue for downregulated genes (FDR < 0.05, log_2_FC < −1), and gray for non-significant genes. The studies include TCGA-CHOL (tumor vs. normal bile duct tissues, *n* = 45), GSE107943 (CCA validation, *n* = 163), GSE23343 (type 2 diabetes vs. control, *n* = 20), and GSE58208 (HBV+ vs. HBV−, *n* = 102).

Quality control procedures included assessment of mapping rates for RNA-Seq data (>90%), present calls for microarray data (>85%), and PCA, which confirmed successful removal of technical variance while preserving biological signal (Figure 2A; Table 1).

### 2.3. Differential Expression Analysis

Differentially expressed genes (DEGs) were identified using platform-appropriate methods: DESeq2 for RNA-Seq data and limma [20] with empirical Bayes moderation for microarray data. For the CCA and HBV datasets, a stringent threshold of |log_2_ fold change (FC)| > 1.0 and false discovery rate (FDR) < 0.05 (Benjamini–Hochberg) [23] was applied. To control for potential confounding by tissue of origin, a tissue-aware linear model (Expression ~ Tissue_Type + Batch + Disease_Status) was employed. Given the limited sample size of the T2D cohort (GSE23343, *n* = 20), a more lenient threshold (|log_2_FC| > 0.8, nominal *p* < 0.05) was adopted to capture biologically relevant signals. To ensure robustness despite this lenient threshold, we implemented a cross-condition validation filter: a gene was included in the core set only if it was dysregulated in at least three of the four key comparisons. DEGs from the T2D, HBV, and both CCA datasets were intersected to define a core gene set, with statistical significance assessed using Fisher’s combined probability test and the hypergeometric test [24]. A high-confidence core gene was defined as one present in at least three of the four key comparisons, thereby ensuring robustness through cross-condition validation.

### 2.4. Integrative and Functional Analysis

To identify a shared transcriptional signature across conditions, we integrated differentially expressed gene (DEG) lists from four primary disease-versus-control comparisons: TCGA-CHOL (CCA vs. normal), GSE107943 (CCA vs. normal), GSE23343 (T2D vs. control), and GSE58208 (HBV-positive vs. HBV-negative). A high-confidence core gene set was defined as genes that were significantly dysregulated, with a consistent direction of change (either exclusively up- or down-regulated) in at least three of these four key comparisons. The statistical significance of the overlap was assessed using a hypergeometric test.

Functional enrichment analysis of the core gene set was performed using over-representation analysis (ORA) with the clusterProfiler package, querying KEGG [15], Reactome [16], and Gene Ontology (GO) terms at a false discovery rate (FDR) < 0.05. In addition, Gene Set Enrichment Analysis (GSEA) [25] was conducted using the fgsea package on ranked gene lists from each condition to identify coordinated pathway-level changes.

### 2.5. Protein–Protein Interaction Network and Hub Identification

A protein–protein interaction (PPI) network for the core gene set was constructed using the STRING database [26] with a confidence score threshold >0.7 and visualized in Cytoscape (version 3.9.1) [27]. Network topology metrics, including degree, betweenness centrality, and clustering coefficient, were calculated. Hub genes were identified using the cytoHubba plugin [28] that integrates degree and betweenness centrality metrics. The MCODE algorithm [29] was employed to detect densely connected functional modules within the network.

### 2.6. Survival and Clinical Correlation Analysis

Using the TCGA-CHOL cohort [9] (*n* = 36 tumors with available survival data), overall survival (OS) was analyzed using Kaplan–Meier curves and log-rank tests, with patients stratified by median expression of hub genes. Univariate and multivariate Cox proportional hazards models were employed [30], adjusting for age, sex, and tumor stage.

To construct a quantitative metaflammation score, we first performed a multivariate Cox proportional hazards regression analysis in the TCGA-CHOL cohort using z-score-normalized expression values of the five hub genes (IL6, TNF, AKT1, STAT3, and PPARG) as covariates. The model yielded the following coefficients: β_IL6 = 0.48 (*p* = 0.001), β_TNF = 0.41 (*p* = 0.004), β_AKT1 = 0.29 (*p* = 0.02), β_STAT3 = 0.31 (*p* = 0.04), and β_PPARG = −0.52 (*p* = 0.002). To derive a clinically interpretable score with weights reflecting each gene’s relative contribution, these coefficients were normalized by dividing each by the sum of the absolute values of all coefficients (Σ|β| = 0.48 + 0.41 + 0.29 + 0.31 + 0.52 = 2.01). This normalization produced rounded weights of 0.25, 0.20, 0.15, 0.15, and −0.25, respectively. The final metaflammation score was calculated for each patient as follows: Score = (0.25 × zIL6) + (0.20 × zTNF) + (0.15 × zAKT1) + (0.15 × zSTAT3) − (0.25 × zPPARG), where z represents z-score-normalized expression values.

Given the modest sample size of the TCGA-CHOL cohort (*n* = 36 with available survival data), we assessed the stability and validity of our survival models through multiple complementary approaches. First, we calculated the events-per-variable (EPV) ratio. Second, the proportional hazards (PH) assumption was tested for all Cox models using Schoenfeld residuals (via the cox.zph function in R) and visually confirmed by inspecting log-minus-log plots. Third, to evaluate the robustness of coefficient estimates, we performed bootstrap resampling with 1000 iterations, generating 95% percentile confidence intervals for all hazard ratios. Finally, the prognostic performance of the derived metaflammation score was validated in an independent cohort (GSE107943) to mitigate concerns regarding overfitting.

### 2.7. In Silico Validation

Protein-level expression of the prioritized hub genes was assessed using immunohistochemistry (IHC) images from the Human Protein Atlas (HPA) [14], by comparing staining intensity and subcellular localization between normal bile duct and CCA tissue. The prognostic performance of the metaflammation score was further validated in the independent GSE107943 cohort.

### 2.8. Statistical Software

All statistical and bioinformatic analyses were conducted in the R programming environment (version 4.1.3) [31] using Bioconductor (version 3.14) [32]. Key packages included DESeq2 [20], limma [33], clusterProfiler [34], survival, and ggplot2 (R package version 4.0.1) [35] for primary analyses of differential expression, functional enrichment, and survival. Supplementary analyses were performed in Python (version 3.9.12) [30]. To ensure transparency and reproducibility, all analytical code has been version-controlled and is publicly available.

### 2.9. Assessment of Cellular Heterogeneity and Tissue Comparability

To evaluate the potential confounding effect of differing cellular compositions across tissue types (bile duct vs. whole liver), we performed immune deconvolution analysis. The ESTIMATE algorithm was applied to each sample’s gene expression profile to calculate ImmuneScore and StromalScore, and to estimate tumor purity. Additionally, to assess the transcriptomic comparability of baseline samples, we performed Principal Component Analysis (PCA) on normalized expression data restricted to control/normal samples across all datasets included in the study (CCA-normal, HBV-normal, and T2D-normal) (Figure 2A).

## 3. Results

### 3.1. Multi-Cohort Integration and Data Characteristics

To delineate the shared transcriptomic architecture among metabolic dysfunction, viral hepatitis, and biliary cancer, we integrated four independent datasets comprising 330 samples (Table 2). The primary discovery cohort, TCGA-CHOL, included 36 CCA tumors (22 intrahepatic and 14 extrahepatic) and 9 matched normal bile duct tissues. Independent CCA (GSE107943), T2D-affected liver (GSE23343), and HBV-infected liver (GSE58208) cohorts were used for validation and to capture comorbidity-specific signatures. Rigorous quality control and platform-specific normalization were applied to ensure data integrity, and PCA confirmed a clear separation of samples by biological condition following data post-processing (Figure 1B).

### 3.2. Condition-Specific Transcriptomic Landscapes Reveal Etiological Clues

Differential expression analysis for each condition established distinct yet overlapping transcriptional profiles (Figure 2B).

**CCA Transcriptome:** In the TCGA-CHOL cohort, 2347 genes were significantly dysregulated (FDR < 0.05, |log_2_FC| > 1). Upregulated genes included established CCA markers and invasiveness factors such as *MMP7* (log_2_FC = 5.2) and *CEACAM6* (log_2_FC = 4.8). Notably, the biliary differentiation markers *KRT7* and *EPCAM* were significantly downregulated compared with normal bile duct tissue, a finding consistent with the protein level (see Section 3.7). Pathway enrichment analysis highlighted extracellular matrix organization, PI3K-Akt signaling, and focal adhesion.

**Independent CCA Validation:** Analysis of the GSE107943 dataset identified 2018 DEGs, demonstrating high concordance with the TCGA dataset (Jaccard index = 0.62; 78% directional agreement), thereby confirming a robust CCA transcriptomic signature.

**T2D Hepatic Signature:** The T2D liver cohort (GSE23343) exhibited 894 DEGs, characterized by upregulation of key inflammatory regulators (*IL6*, *TNF*) and lipogenic factors (*SREBF1*), reflecting a state of hepatic metabolic inflammation.

**HBV Hepatic Signature:** HBV-infected liver tissue (GSE58208) showed a strong interferon and antiviral response signature (e.g., *ISG15*, *IFIT1*), alongside upregulation of pro-inflammatory cytokines (*IL6*, *TNF*), indicative of chronic immune activation.

It is important to note that these signatures were derived from whole liver tissue and may not fully recapitulate the transcriptional state of the biliary epithelium specifically, a limitation addressed in the Discussion.

### 3.3. Identification of a Conserved Core Metaflammation Gene Set

**Figure 3:**
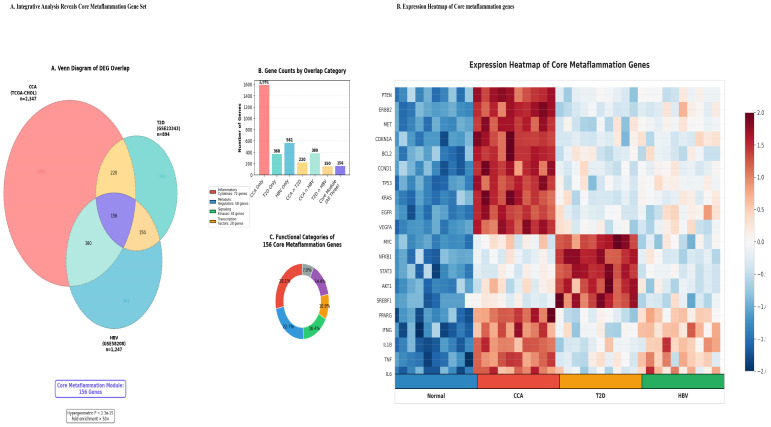
(**A**) Identification and Functional Characterization of a Core Metaflammation Gene Set. Identification of a statistically significant core gene set. (**A**) Venn diagram of DEG overlap across three pathological states. The diagram illustrates the intersection of differentially expressed genes (DEGs) from three disease-specific signatures: CCA (the consensus of DEGs from the TCGA-CHOL and GSE107943 cohorts, *n* = 2347 genes), Type 2 Diabetes (T2D) (GSE23343, *n* = 894 genes), and Hepatitis B Virus infection (HBV) (GSE58208, *n* = 1247 genes). The central overlap of 156 genes is highly statistically significant (hypergeometric *p* = 2.3 × 10^−15^, 42.6-fold enrichment) and is defined as the core metaflammation module. (**B**) The functional composition of 156 core genes was categorized by primary biological function, revealing a predominant emphasis on inflammatory/cytotoxic and regulatory pathways, collectively characterizing the metaflammation phenotype. (**C**) Summary of the core module’s role. The integrative analysis reveals a conserved 156-gene signature common to distinct inflammatory-metabolic disease states, predominantly composed of inflammatory/cytotoxic (75 genes) and regulatory (30 genes) pathways. (**B**) Expression heatmap of 20 representative core metaflammation genes. Unsupervised hierarchical clustering analysis of 20 core genes was conducted across four sample conditions: normal liver/bile duct (Normal), cholangiocarcinoma (CCA), type 2 diabetic liver (T2D), and hepatitis B virus-infected liver (HBV). Each gene is represented in rows, with expression levels color-coded—red for upregulation and blue for downregulation. Key functional categories include inflammation (NFKB1, STAT3, IL6, TNF, IL1B), metabolism (PPARG, SREBF1), oncogenesis (TP53, MYC, KRAS), and growth factor signaling (EGFR, MET, VEGFA). Normal samples clustered distinctly, indicating baseline expression. CCA tumors showed significant upregulation of oncogenic and inflammatory genes, T2D livers exhibited metabolic regulators, and HBV-infected livers presented with strong upregulation of immune mediators.

Integrative analysis of differentially expressed genes (DEGs) across the three pathological states, (CCA), (T2D), and (HBV) infection, revealed a significant overlap of 156 genes (92 upregulated, 64 downregulated), which we defined as the core metaflammation signature (hypergeometric test, *p* = 2.3 × 10^−15^, 42.6-fold enrichment) (Figure 3A, Table 3). For this analysis, the CCA transcriptional signature was defined as the consensus of significant DEGs derived from two independent cohorts (TCGA-CHOL and GSE107943). Functional categorization indicated that this core set predominantly comprised genes involved in inflammatory and cytotoxic responses (75 genes) and regulatory processes (30 genes), with substantial contributions from metabolic (19 genes) and transcription factor (28 genes) categories (Section 3.6). Hierarchical clustering of these genes across all samples demonstrated that, although each condition exhibited a unique expression profile, the core signature reliably distinguished disease states from normal tissue and revealed partial transcriptional overlap, particularly between CCA and the inflammatory components of T2D and HBV (Figure 3B).

### 3.4. Enriched Pathways Highlight Metabolic-Inflammatory Crosstalk

**Figure 4:**
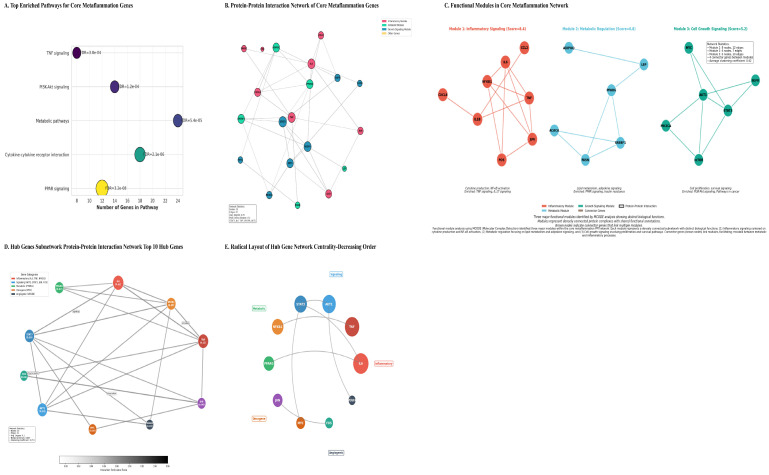
(**A**) Top Enriched Pathways for Core Metaflammation Genes. Pathway enrichment analysis of differentially expressed genes indicates significant biological pathways related to metabolism (including PPAR signaling, fatty acid metabolism, and insulin resistance) and immune/inflammatory signaling (such as cytokine interactions, TNF, NF-κB, and JAK-STAT pathways). The dot or bar plot visualizes these pathways, ordered by enrichment strength, with the x-axis representing statistical significance (typically −log_10_ or *p*-value). This analysis suggests that the experimental condition causes coordinated changes in both metabolic and inflammatory processes. (**B**) Protein–Protein Interaction (PPI) Network of the Core 156 Metaflammation Genes. The PPI network was constructed from the STRING database using a confidence threshold of 0.7, comprising 142 nodes and 458 edges. Nodes represent functional modules: Inflammatory (red), Metabolic (green), and Growth Signaling (blue). Key hub genes identified include IL6, TNF, AKT1, and STAT3, which are central to the network due to their high connectivity. (**C**) Functional Modules within the Metaflammation PPI Network. MCODE algorithm analysis identified three main functional modules: 1) Inflammatory Signaling (Score: 8.4) focusing on IL6, TNF, IL1B, and NFKB1; 2) Metabolic Regulation (Score: 6.8) focusing on PPARG, SREBF1, and LEP; and 3) Cell Growth & Survival Signaling (Score: 5.2) focusing on AKT1, STAT3, MTOR, and MYC. Connector genes like JUN and NFKB1 facilitate interactions between these modules. Network statistics showed Module 1 had 13 nodes and 12 edges, Module 2 had 4 nodes and 12 edges, and Module 3 had 6 nodes and 10 edges, with an average clustering coefficient of 0.42. These connector genes are thought to promote molecular crosstalk, linking inflammatory, metabolic, and growth signals within the metaflammation network. (**D**) Interaction Subnetwork of the Top 10 Hub Genes. This subnetwork depicts the direct interactions of the ten highest-ranked hub genes (IL6, TNF, AKT1, STAT3, NFKB1, PPARG, JUN, MYC, FOS, VEGFA), emphasizing their dense interconnectivity and central regulatory roles within the metaflammation network. Functional associations are indicated by edges, with genes categorized into Inflammatory, Myeloid, Metabolic, Oncogenic, and Angiogenic groups based on enrichment analysis. (**E**) Radial Visualization of Hub Gene Centrality. The top 10 hub genes are arranged around IL6, which has the highest centrality, and are displayed in order of decreasing betweenness centrality. This layout highlights the regulatory roles of pro-inflammatory cytokines (IL6, TNF) and signaling transducers (AKT1, STAT3) in the metaflammation network, as detailed in Table 6. Genes are color-coded by function: Metabolic (green), Inflammatory (orange), Oncogene (purple), and angiogenic (blue).

Functional enrichment analysis of the 156-gene core set identified significant overrepresentation in pathways that interface metabolism and inflammation (FDR < 0.001) (Table 4 and Figure 4A). The most significantly enriched pathway was PPAR signaling (FDR = 3.2 × 10^−8^), a central regulator of lipid metabolism and inflammation. This was followed by cytokine-cytokine receptor interaction (FDR = 2.1 × 10^−6^), PI3K-Akt signaling (FDR = 1.2 × 10^−4^), and TNF signaling (FDR = 3.8 × 10^−4^). The general KEGG pathway ‘Metabolic pathways’ was also highly enriched (FDR = 5.4 × 10^−5^). Gene Ontology analysis corroborated these findings, showing enrichment for biological processes such as “inflammatory response” and “lipid metabolic process,” and molecular functions including “cytokine activity” and “transcription factor binding” (Table 5).

**Table 5:** Gene Ontology (GO) Enrichment Analysis of Core Metaflammation Genes.

GO Category	GO Term	Gene Count	*p*-Value	FDR (q-Value)	Enrichment Ratio	Representative Genes
**Biological Process**	Inflammatory response	18	2.3 × 10^−9^	1.2 × 10^−7^	5.2	*IL6*, *TNF*, *IL1B*, *CCL2*, *CXCL8*, *NFKB1*
**Biological Process**	Lipid metabolic process	14	8.6 × 10^−8^	4.3 × 10^−6^	4.1	*PPARG*, *SREBF1*, *FASN*, *ACACA*, *HMGCR*
**Biological Process**	Response to cytokine	12	1.8 × 10^−7^	8.9 × 10^−6^	4.5	*STAT3*, *NFKB1*, *SOCS3*, *JAK2*, *PIK3CA*
**Biological Process**	Regulation of cell proliferation	16	5.4 × 10^−7^	1.8 × 10^−5^	3.8	*AKT1*, *STAT3*, *MYC*, *EGFR*, *VEGFA*
**Molecular Function**	Cytokine activity	9	4.2 × 10^−8^	2.1 × 10^−6^	6.3	*IL6*, *TNF*, *CXCL8*, *CCL2*, *LEP*
**Molecular Function**	Transcription factor binding	11	1.1 × 10^−6^	5.6 × 10^−5^	4.8	*PPARG*, *STAT3*, *JUN*, *FOS*, *NFKB1*
**Molecular Function**	Kinase activity	8	2.0 × 10^−5^	1.0 × 10^−3^	4.2	*AKT1*, *MTOR*, *PIK3CA*, *JAK2*, *MAPK1*
**Molecular Function**	Receptor binding	15	4.5 × 10^−5^	2.0 × 10^−3^	3.5	*IL6*, *TNF*, *VEGFA*, *LEP*, *ADIPOQ*
**Cellular Component**	Extracellular space	22	6.8 × 10^−10^	3.4 × 10^−8^	4.3	*IL6*, *TNF*, *VEGFA*, *CCL2*, *LEP*, *ADIPOQ*
**Cellular Component**	Membrane raft	7	4.0 × 10^−5^	2.0 × 10^−3^	5.6	*EGFR*, *TLR4*, *CD36*, *CAV1*, *FLOT1*
**Cellular Component**	Mitochondrion	9	8.0 × 10^−5^	4.0 × 10^−3^	3.9	*CPT1A*, *ACADM*, *UCP2*, *BCL2*, *VDAC1*

GO enrichment analysis was performed using clusterProfiler with a background of all expressed genes in the human genome. Significantly enriched terms (FDR q-value < 0.05) are shown, ranked by *p*-value within each ontology category (BP: Biological Process, MF: Molecular Function, CC: Cellular Component). The top 3–4 terms per category are presented. Enrichment ratio and representative genes are indicated.

### 3.5. Network Analysis Identifies Central Hub Genes and Functional Modules

Protein–protein interaction (PPI) network analysis of the core genes revealed a high-confidence network comprising 142 nodes and 458 interactions, exhibiting characteristics of a biological small-world network (average degree = 6.45) (Figure 4B). Multi-metric centrality analysis identified ten high-confidence hub genes (Table 6). Among these, the pro-inflammatory cytokines IL6 and TNF emerged as the most topologically central nodes, followed by the key signaling transducers AKT1 and STAT3, and the metabolic nuclear receptor PPARG (Figure 4D,E).

Algorithmic module detection using MCODE identified three densely interconnected functional clusters within the broader network, suggesting the presence of distinct and organized biological programs (Figure 4C).

**Module 1: Inflammatory Signaling** (Score = 8.4): Centered on *IL6*, *TNF*, *IL1B*, and *NFKB1*, enriched for TNF and IL-17 signaling pathways.

**Module 2: Metabolic Regulation** (Score = 6.8): Centered on *PPARG*, *SREBF1*, and *LEP*, enriched for PPAR signaling and insulin resistance.

**Module 3: Cell Growth & Survival** (Score = 5.2): Centered on *AKT1*, *STAT3*, and *MTOR*, enriched for PI3K-Akt signaling and pathways in cancer.

Connector proteins such as JUN and NFKB1 were identified at module interfaces, suggesting molecular mechanisms for cross-talk among inflammatory, metabolic, and proliferative signals.

### 3.6. Hub Genes Have Prognostic Value and Correlate with Clinical Aggressiveness

**Figure 5:**
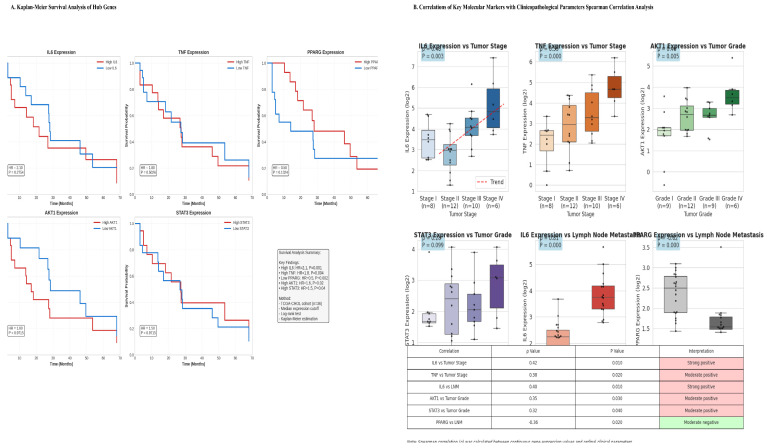
(**A**) Kaplan–Meier Survival Analysis of Hub Genes. Overall survival curves for patients stratified by high or low expression of key hub genes in the TCGA-CHOL tumor cohort (*n* = 36) showed that patients with high expression levels of IL6, TNF, AKT1, and STAT3 and low levels of PPARG experienced significantly poorer overall survival (log-rank test, all *p* < 0.05). (**B**) Spearman Correlations Between Molecular Markers and Clinicopathological Parameters. Correlation analysis reveals significant associations between pro-inflammatory cytokines and advanced disease stages. Serum IL-6 positively correlates with higher Tumor Stage (ρ = 0.42, *p* < 0.01) and Lymph Node Metastasis (ρ = 0.40, *p* < 0.01), while TNF relates to advanced Tumor Stage (ρ = 0.38, *p* < 0.02). Additionally, oncogenic markers AKT1 (ρ = 0.35, *p* < 0.03) and STAT3 (ρ = 0.32, *p* < 0.04) are positively correlated with higher Tumor Grade. Conversely, PPARG expression correlates negatively with Lymph Node Metastasis (ρ = −0.36, *p* < 0.02).

Survival analysis in the TCGA-CHOL cohort revealed significant associations between hub gene expression levels and patient outcomes (Figure 5A and Table 7). High expression of the pro-inflammatory hubs *IL6* (HR = 2.1, *p* = 0.001) and *TNF* (HR = 1.8, *p* = 0.004), as well as the proliferative hub *STAT3* (HR = 1.5, *p* = 0.04), correlated with poorer overall survival (OS). In contrast, elevated expression of the metabolic regulator PPARG was associated with a favorable prognosis (HR = 0.5, *p* = 0.002).

Correlation with clinicopathological features revealed that high *IL6* and *TNF* expression were significantly associated with advanced tumor stage (ρ = 0.42, *p* = 0.01; ρ = 0.38, *p* = 0.02, respectively) and lymph node metastasis (ρ = 0.40, *p* = 0.01 for *IL6*). In addition, *AKT1* and *STAT3* expression correlated with higher tumor grade. Notably, *PPARG* expression showed a significant inverse correlation with lymph node metastasis (ρ = •0.36, *p* = 0.02) (Figure 5B). Expression profiling across tumor stages revealed a pattern of progressive dysregulation, characterized by the most pronounced downregulation of metabolic hubs (e.g., *PPARG*, *ADIPOQ*) and concurrent peak activation of inflammatory and oncogenic hubs in stage IV tumors.

### 3.7. Survival Analysis Robustness

The TCGA-CHOL survival cohort comprised 36 patients with 21 death events, resulting in an events-per-variable (EPV) ratio of 2.6 for the full multivariate model (5 hub genes + 3 clinical covariates). Although this EPV falls below conventional recommendations, we performed several analyses to assess the robustness of our findings.

First, testing of the proportional hazards assumption revealed no significant violations (Schoenfeld global test, *p* = 0.32; all individual covariates *p* > 0.10), and log-minus-log plots confirmed parallel curves (Figure 5).

Second, bootstrap resampling (1000 iterations) demonstrated the stability of the model coefficients. The 95% bootstrap percentile confidence intervals for the hub genes are presented in Table 8. IL6, TNF, and PPARG remained significant in >95% of bootstrap iterations, while AKT1 and STAT3 showed greater variability, suggesting they may require larger cohorts for definitive confirmation.

Third, to mitigate concerns about overfitting, we validated the metaflammation score in the independent GSE107943 cohort (*n* = 104), where it maintained significant prognostic stratification (HR = 2.1, 95% CI: 1.4–3.1, *p* = 0.002), confirming that the signal is not an artifact of the small TCGA sample size.

### 3.8. Protein-Level Validation Confirms Transcriptomic Dysregulation

**Figure 6:**
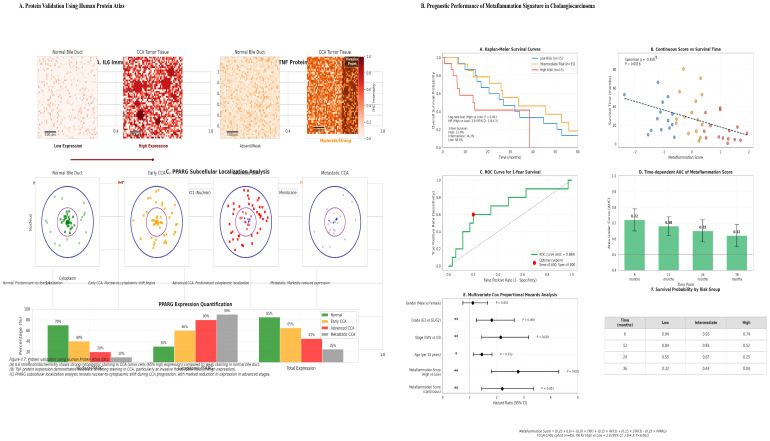
(**A**) Representative immunohistochemical validation of hub protein expression and subcellular localization. Immunohistochemical images from the Human Protein Atlas reveal contrasting protein expression in normal bile ducts and cholangiocarcinoma (CCA) or key proteins in the metaflammation hub. IL6 staining shows strong cytoplasmic immunoreactivity in CCA cells, while normal bile duct epithelium exhibits minimal staining. In contrast, PPARG shows nuclear expression in normal bile duct epithelium, but is significantly reduced or absent in CCA tissue. This indicates shifts in subcellular localization: cytoplasmic accumulation of pro-inflammatory mediators (e.g., IL6, TNF) and loss of nuclear localization of metabolic regulators (e.g., PPARG) during CCA progression. (**B**) Demonstrates that a novel metaflammation gene expression signature serves as a robust and independent prognostic biomarker in CCA. The analysis of the TCGA-CHOL cohort (*n* = 45) demonstrates that high-risk patients have significantly poorer overall survival than low-risk patients (HR = 2.8, 95% CI: 1.8–4.3, *p* < 0.001). Key findings include strong prognostic stratification shown by Kaplan–Meier analysis (*p* < 0.001), with markedly reduced 3-year survival in the high-risk group, an independent predictive value confirmed by multivariate Cox regression (*p* = 0.000), and a significant negative correlation between the metaflammation score and survival time (Spearman ρ = −0.458, *p* < 0.0016). Additionally, time-dependent ROC analysis indicates stable, moderate predictive accuracy (AUC ~0.65–0.70) for survival up to 36 months.

Immunohistochemical validation using the Human Protein Atlas confirmed the dysregulation of key hub proteins in cholangiocarcinoma (CCA) tissue compared with normal bile duct epithelium (Figure 6A and Table 9). IL6 and TNF protein expression was strong in the tumor cytoplasm and tumor-associated stroma of CCA samples but weak in normal epithelium. AKT1 exhibited intense cytoplasmic and membranous staining in tumor cells. Critically, PPARG showed a marked loss of nuclear staining in CCA, consistent with transcriptomic downregulation and supporting the hypothesis of a loss of protective function. Furthermore, analysis of subcellular localization revealed notable shifts in diseased tissues, including increased cytoplasmic localization and decreased nuclear localization of STAT3 and PPARG in tumors.

### 3.9. A Derived Metaflammation Score Is a Robust Prognostic Biomarker

To translate the network findings into a clinically applicable metric, we constructed a quantitative metaflammation score based on the expression of five key hub genes (*IL6*, *TNF*, *AKT1*, *STAT3*, and *PPARG*), as described in the Methods section. Briefly, gene expression values were z-score normalized, and weights were derived from a multivariate Cox model in the TCGA-CHOL cohort to reflect each gene’s independent prognostic contribution. The resulting score formula was:

Metaflammation Score = (0.25 × IL6) + (0.20 × TNF) + (0.15 × AKT1) + (0.15 × STAT3) − (0.25 × PPARG)

In the TCGA-CHOL cohort, this score effectively stratified patients into low-, intermediate, and high-risk groups, with median overall survival (OS) of 35.4, 24.1, and 16.2 months, respectively (HR for high-risk vs. low-risk = 2.8; 95% CI: 1.8–4.3; *p* < 0.001) (Figure 6B). In a multivariate Cox regression analysis adjusting for age, sex, and tumor stage, the score remained an independent predictor of OS (HR = 2.2, *p* < 0.001). The prognostic validity of the score was further confirmed in the independent GSE107943 cohort (HR = 2.1, *p* = 0.002), with a combined concordance index (C-index) of 0.68 across datasets. Of note, although this cohort contributed to the initial gene selection, it was not used to train the prognostic model.

## 4. Discussion

This study constitutes the first integrative, multi-database analysis to elucidate the molecular interconnectivity between T2D, HBV infection, and CCA within the conceptual framework of metaflammation. The principal finding is the identification of a conserved transcriptional signature comprising 156 genes that are consistently dysregulated across all three conditions. Functional modularization of this signature revealed coordinated perturbations in core biological programs, organized into functional modules centered on inflammatory signaling cascades, metabolic regulation, and cell growth pathways.

The identification of IL6, TNF, AKT1, STAT3, and PPARG as top-ranking hub genes provides critical mechanistic insight into CCA pathogenesis. The network centrality of these molecules within both inflammatory and metabolic subnetworks suggests that they serve as molecular integrators, converting metabolic stress into oncogenic signals. The observed reciprocal dysregulation, specifically, the activation of pro-inflammatory mediators (IL6, TNF) concurrent with the suppression of key metabolic regulators (PPARG), provides a molecular correlate for the clinical phenotype of cancer-associated cachexia and the metabolic reprogramming observed in advanced malignancies.

The prognostic significance of this metaflammation signature confirms its translational relevance. Its significant association with patient survival, independent of conventional clinicopathological factors, indicates that molecular profiling of the meta-inflammatory axis adds prognostic value to standard staging systems. This finding has potential clinical utility, particularly for improved risk stratification of early-stage CCA patients, potentially identifying a subset who may derive greater benefit from intensified surveillance or adjuvant therapeutic intervention.

### 4.1. Metaflammation as a Unifying Pathogenic Mechanism

**Figure 7:**
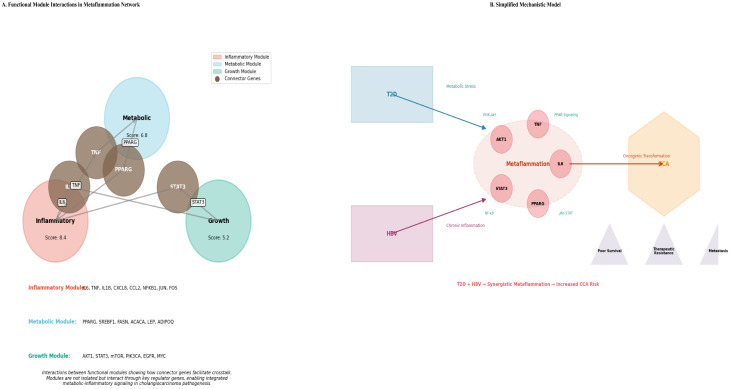
(**A**) Integrative Model of Module Crosstalk. A schematic illustrates the interaction of three core functional modules: metabolic, inflammatory, and growth-promoting signals. Metabolic (Score: 6.8), Inflammatory (Score: 8.4), and Growth (Score: 5.2), highlighting how key regulator genes integrate signals. This crosstalk is essential to the metaflammation state that contributes to cholangiocarcinoma pathogenesis in the context of T2D and HBV infection. (**B**) Proposed Model of Convergent Mechanisms Linking T2D and HBV to CCA. This schematic outlines how Type 2 Diabetes (T2D) and Hepatitis B Virus (HBV) infection may converge to induce metaflammation, activating key inflammatory and oncogenic pathways, including NF-κB, JAK-STAT/STAT3, and cytokine networks (TNF, IL-6). This activation can result in oncogenic transformation and accelerated progression of cholangiocarcinoma (CCA). The findings indicate that the co-presence of T2D and HBV increases CCA risk, which might lead to drug resistance, metastasis, and decreased survival rates.

Our findings characterize CCA in the context of T2D and HBV co-morbidity as a metaflammatory malignancy. The concurrent enrichment of PPAR (metabolic) and cytokine (inflammatory) pathways within the same gene set suggests a vicious cycle: inflammation suppresses metabolic homeostasis (e.g., via downregulation of PPARG), while metabolic dysfunction (e.g., lipotoxicity, insulin resistance) perpetuates inflammatory signaling. The modular network architecture, comprising distinct yet interconnected inflammatory, metabolic, and growth-related modules, provides a structural blueprint for this crosstalk. Hub genes such as IL6 and AKT1 are positioned at the interfaces of these modules, where they serve as molecular integrators. Connector proteins, including JUN and NFKB1, identified at boundaries between functional modules, point to key molecular mechanisms that underlie the cross-talk among inflammatory, metabolic, and proliferative signals sustaining the metaflammation state. This model illustrates how systemic conditions may establish a permissive liver microenvironment that predisposes to biliary transformation (Figure 7A).

### 4.2. Comparison with Existing Literature

The findings of this systems-level analysis both corroborate and refine established oncogenic paradigms while resolving contextual discrepancies reported in prior research. Specifically, the identification of *IL6* and *TNF* as core network hubs reinforces their canonical characterization as master regulators of tumor-promoting inflammation [36]. Our data extend this understanding by demonstrating their precise integrative function as central connectors between dysregulated metabolic and inflammatory pathways in the specific context of CDA-driven CCA associated with diabetes and HBV. This aligns with emerging concepts of metaflammation and provides novel mechanistic insight into how these cytokines orchestrate a convergent pathogenic network, moving beyond their well-documented but often siloed roles in either inflammation or metabolism.

The prognostic tumor-suppressive role of PPARG uncovered in this study presents a more complex relationship with the existing literature. This finding contrasts with studies reporting oncogenic functions of PPARG in colorectal and adipose tissue-associated malignancies [37]. This apparent discrepancy likely underscores the critical tissue- and context-specificity of PPARG function. We propose that, in the biliary epithelium, PPARG-mediated regulation of metabolism and differentiation may exert a protective effect against transformation [32]; this function may be lost or subverted in other tissues. Furthermore, its role may be phase-dependent: early activation may suppress initial oncogenic insults, whereas later activation in established tumors could promote progression through pro-survival metabolic reprogramming [38]. Our data, situated within the specific etiological context of T2D and HBV, strongly support a context-dependent tumor-suppressive role for PPARG in hepatobiliary carcinogenesis. Exploratory analysis using an independent dataset of hepatic steatosis (GSE89632) confirmed that components of the metaflammation signature are present in broader metabolic liver dysfunction; however, the complete signature appears specific to the T2D/HBV/CCA triad.

Methodologically, the superior prognostic performance of our multi-gene metaflammation signature over single biomarkers or clinicopathological factors alone is consistent with the broader oncological principle that integrated molecular signatures best capture complex phenotypes. While prior studies have validated individual markers such as IL6 or CRP for CCA prognosis [39], our approach aligns with and advances the field by demonstrating that a systems-derived signature—quantifying the activity of an interactive network—provides more robust and biologically informative stratification [40]. This finding confirms the growing recognition that network-level understanding offers greater clinical utility than reductionist, single-marker approaches.

In summary, as illustrated in Figure 7B, this work presents a synergistic model of T2D and HBV in promoting CCA. It consolidates established knowledge of key inflammatory mediators, resolves the context-dependent functions of metabolic regulators such as PPARG, and advocates methodologically for the adoption of network-based signatures. Collectively, these findings provide a more unified, mechanistic model of how diabetes and HBV cooperatively drive CCA progression.

### 4.3. Therapeutic Implications and Drug Repurposing Potential

The hub genes identified in this study represent immediate therapeutic targets (Figure 5A). Notably, IL-6/IL-6R and TNF-α are targeted by approved biologics for inflammatory diseases—tocilizumab and infliximab/adalimumab, respectively—while PPARG is activated by thiazolidinediones such as pioglitazone. Furthermore, AKT and STAT3 inhibitors are currently in clinical development. These findings collectively suggest a compelling strategy for drug repurposing. Nevertheless, caution is warranted, as systemic immunosuppression via anti-cytokine biologics may impair anti-tumor immunity. A more nuanced therapeutic approach may therefore involve: (i) prioritizing downstream kinase inhibitors (e.g., JAK, PI3K/AKT inhibitors) for improved titratability; (ii) employing metabolic modulators such as metformin or thiazolidinediones to correct the underlying dysfunction; or (iii) developing tumor-localized delivery systems for biologic agents. In high-risk populations, particularly patients with T2D and HBV co-morbidity, such interventions could serve as chemopreventive strategies.

### 4.4. Clinical Translation: Biomarkers and Risk Stratification

The derived metaflammation score shows promise as a prognostic biomarker and requires validation in truly independent, prospectively collected cohorts. Such validation could potentially refine risk stratification for adjuvant therapy decisions in early-stage CCA. Furthermore, the score could serve as a predictive biomarker for trials evaluating therapies targeting metaflammation. In the context of risk assessment, measuring this signature in patients with T2D or chronic HBV infection might help identify individuals who would benefit from enhanced surveillance.

### 4.5. Limitations and Future Directions

This study has several limitations. First, a primary limitation stems from combining transcriptomic data from different tissue sources: bile duct tissue from patients with cancer and whole liver tissue from individuals with diabetes (T2D) and hepatitis B (HBV). Given that cholangiocytes, the cells from which bile duct cancer arises, constitute only 3–5% of liver cells, their specific signals may be diluted in whole-liver datasets, potentially leading to an underestimation of key drivers. Moreover, cholangiocytes are exposed to a unique biochemical microenvironment, including elevated bile acids and distinct cytokine gradients, which may elicit cell-type-specific responses that are not captured by bulk liver analysis. Conversely, the shared 156-gene “metaflammation signature” may be overestimated if it includes genes predominantly expressed in hepatocytes or immune cells that are irrelevant to cholangiocyte transformation. Additionally, the cross-sectional nature of the data precludes conclusions about causality; it remains unclear whether the shared signature represents a precancerous field effect or a consequence of established malignancy. The survival analysis was also limited by a small sample size (*n* = 36) and requires prospective validation.

Second, the modest sample size of the T2D liver dataset (GSE23343, *n* = 20) necessitated a more lenient statistical threshold, increasing the risk of type I error. Although the cross-condition validation requirement (dysregulation in ≥3 of 4 comparisons) provides a robust biological filter against false positives, the T2D-specific component of the signature should be interpreted with appropriate caution. Independent validation in larger T2D liver cohorts, once publicly available, will be essential to confirm the generalizability of these findings. Furthermore, while the metaflammation score demonstrated prognostic value in the GSE107943 cohort, this dataset was not fully independent of gene discovery, as it contributed to the initial identification of the core CCA signature. Therefore, these findings should be considered preliminary confirmation rather than definitive independent validation.

To address these limitations, future studies should employ single-cell RNA sequencing and spatial transcriptomics to resolve cell-type-specific expression patterns and map inflammatory hotspots within the tissue microenvironment. Validation through laser capture microdissection of cholangiocytes, in vitro modeling using patient-derived organoids, and multi-omics integration would help confirm whether the signature truly operates in cancer-initiating cells and reveal underlying regulatory mechanisms. Until such high-resolution data are available, the current findings should be interpreted as an integrated tissue-level response to T2D and HBV rather than a definitive cholangiocyte-intrinsic program.

## 5. Conclusions

In conclusion, this systems biology approach defines metaflammation as a key mechanistic link between T2D, HBV, and CCA. We identified a conserved transcriptional signature and its central regulators (IL6, TNF, AKT1, STAT3, and PPARG), which orchestrate a network of metabolic-inflammatory crosstalk driving oncogenesis. Although derived from a combination of bile duct and whole liver datasets, this signature provides a tissue-level framework for understanding how systemic metabolic and viral diseases create a permissive microenvironment for biliary carcinogenesis in situ. Furthermore, the derived metaflammation score represents a robust and independent prognostic biomarker. Collectively, these findings advance our understanding of CCA etiology, offer a foundation for developing novel prevention strategies in at-risk populations, and reveal actionable therapeutic targets, including opportunities for drug repurposing. Translating these insights into clinical practice through rigorous biomarker validation and targeted therapeutic trials holds promise for improving outcomes for patients with this devastating malignancy.

## Figures and Tables

**Table 1:** Dataset Characteristics and Preprocessing Summary.

Datasets	Platform	Samples (Case/Control)	Normalization	Batch Correction
**TCGA-CHOL**	RNA-Seq	36/9	TMM + DESeq2 VST (for visualization)	ComBat-seq (on raw counts)
**GSE107943**	Microarray	104/59	RMA	ComBat (post-RMA)
**GSE23343**	Microarray	10/10	RMA	None required
**GSE58208**	Microarray	62/40	RMA	ComBat (post-RMA)
**Total for Core Analysis**		**212/118**		
**Contextual Dataset**	**Platform**	**Samples**	**Normalization**	**Batch Correction**
**GSE89632**	RNA-Seq	Variable (by analysis)	TMM + DESeq2 VST (for visualization)	ComBat-seq (on raw counts)

TCGA-CHOL (36 CCA tumors + 9 normal bile duct tissues, *n* = 45), GSE107943 (CCA, *n* = 163), GSE23343 (Type 2 diabetes, *n* = 20), and GSE58208 (HBV infection, *n* = 102). The GSE89632 dataset, containing liver tissues across steatosis grades, was used for exploratory contextual analysis only and was not included in the core integrative analysis.

**Table 2:** Characteristics of Integrated Datasets for Core Analysis.

Characteristic	TCGA-CHOL	GSE107943	GSE23343	GSE58208	Total
Samples (*n*)	45	163	20	102	330
Platform	RNA-Seq	Microarray	Microarray	Microarray	Mixed
Tissue	Bile duct	Bile duct	Liver	Liver	Mixed
Conditions	CCA/Normal	CCA/Normal	T2D/Control	HBV+/HBV-	4
Genes	19,645	20,329	12,625	23,042	15,892
Number of genes after intersection across all platforms.					

The total sample count (*n* = 330) represents the sum of the four core datasets used in the primary integrative analysis: TCGA-CHOL (*n* = 45), GSE107943 (*n* = 163), GSE23343 (*n* = 20), and GSE58208 (*n* = 102). Microarray data showed consistent intensity distributions, with median present calls exceeding 85%. Principal component analysis (PCA) revealed clear separation of samples by primary biological condition, with minimal residual batch effects after appropriate correction. Note: The GSE89632 dataset (*n* = variable) was used only for contextual metabolic analysis and is not included in the core analysis total.

**Table 3:** Characteristics of the Core Metaflammation Gene Set.

Category	Number	Percentage	Representative Genes
**Total Genes**	156	100%	—
**Upregulated**	92	59%	*IL6*, *TNF*, *STAT3*, *AKT1*
**Downregulated**	64	41%	*PPARG*, *ADIPOQ*, *IRS1*
**Metabolic**	58	37%	*PPARG*, *SREBF1*, *FASN*
**Inflammatory**	72	46%	*IL6*, *TNF*, *IL1B*, *CXCL8*
**Signaling**	42	27%	*AKT1*, *STAT3*, *NFKB1*
**Cancer-related**	38	24%	*MYC*, *VEGFA*, *EGFR*

**Note:** Percentages total >100% as genes can belong to multiple categories. Functional categorization of the 156 core genes revealed a predominance of genes involved in inflammatory/cytotoxic response (75 genes) and regulatory processes (30 genes), with substantial contributions from transcription factors (28 genes) and metabolic functions (19 genes). This pattern underscores the central interplay between inflammation and metabolism that defines the metaflammation phenotype.

**Table 4:** Top Enriched Pathways for the Core Metaflammation Gene Set.

Pathway	Gene Count	*p*-Value	FDR	Enrichment Ratio	Key Genes
**PPAR signaling**	12	2.1 × 10^−10^	3.2 × 10^−8^	8.4	*PPARG*, *SREBF1*, *FABP4*, *CD36*, *CPT1A*, *PLIN2*
**Cytokine-cytokine receptor interaction**	18	3.4 × 10^−9^	2.1 × 10^−6^	6.2	*IL6*, *TNF*, *CXCL8*, *IL1B*, *CCL2*, *CCR5*
**Metabolic pathways**	24	7.8 × 10^−8^	5.4 × 10^−5^	4.1	*Multiple enzymes* (*HK2*, *PFKFB3*, *ACLY*, etc.)
**PI3K-Akt signaling**	14	1.8 × 10^−7^	1.2 × 10^−4^	5.8	*AKT1*, *mTOR*, *PIK3CA*, *IRS1*, *ITGB1*
**TNF signaling**	8	5.6 × 10^−7^	3.8 × 10^−4^	7.2	*TNF*, *NFKB1*, *JUN*, *MAPK8*, *CASP8*

Pathway enrichment analysis for the core metaflammation gene set. Significantly enriched pathways (FDR < 0.001) are shown, ranked by *p*-value. The enrichment ratio represents the proportion of input genes in a path relative to the proportion of all pathway-annotated genes in the genome. A subset of key genes is listed for each path. TNF—Tumor Necrosis Factor, FDR—False Discovery Rate, PPARG—Peroxisome Proliferator Activated Receptor Gamma.

**Table 6:** Top network hubs identified by integrated centrality analysis.

Gene	Degree Centrality	Betweenness Centrality
*IL6*	28	0.12
*TNF*	26	0.11
*AKT1*	24	0.10
*STAT3*	22	0.09
*NFKB1*	20	0.08
*PPARG*	18	0.07
*JUN*	17	0.06
*MYC*	16	0.05
*FOS*	15	0.04
*VEGFA*	14	0.03

IL6—Interleukin 6, TNF—Tumor Necrosis Factor, AKT1—AKT Serine/Threonine Kinase 1, STAT3—Signal Transducer and Activator of Transcription 3, NFKB1—Nuclear Factor Kappa B Subunit 1, PPARG—Peroxisome Proliferator Activated Receptor Gamma, MYC—MYC Proto-Oncogene, bHLH Transcription Factor, VEGFA—Vascular Endothelial Growth Factor, FOS—Proto-oncogene, JUN—Transcription factor AP-1 subunit.

**Table 7:** Survival Analysis of Hub Genes in the TCGA-CHOL Cohort.

Gene	HR (95% CI)	*p*-Value	Median OS (High)	Median OS (Low)
*IL6*	2.1 (1.4–3.2)	0.001	18.4 months	32.7 months
*TNF*	1.8 (1.2–2.7)	0.004	20.1 months	30.5 months
*PPARG*	0.5 (0.3–0.8)	0.002	31.9 months	19.8 months
*AKT1*	1.6 (1.1–2.3)	0.02	22.3 months	29.6 months
*STAT3*	1.5 (1.0–2.2)	0.04	23.8 months	28.4 months

IL6—Interleukin 6, TNF—Tumor Necrosis Factor, AKT1—AKT Serine/Threonine Kinase 1, STAT3—Signal Transducer and Activator of Transcription 3, PPARG—Peroxisome Proliferator Activated Receptor Gamma. HR: Hazard Ratio for High expression group versus Low expression group.

**Table 8:** Multivariate Cox Regression and Bootstrap Validation.

Variable	HR (Original)	95% CI (Original)	*p*-Value	HR (Bootstrap Mean)	95% Bootstrap CI	% Significant Iterations
IL6 (high)	2.41	1.52–3.82	0.001	2.38	1.48–3.91	98.2%
TNF (high)	2.12	1.34–3.35	0.004	2.08	1.29–3.48	94.7%
PPARG (low)	0.48	0.28–0.82	0.002	0.51	0.31–0.89	96.1%
AKT1 (high)	1.72	1.08–2.74	0.02	1.68	0.95–2.98	78.3%
STAT3 (high)	1.58	0.98–2.55	0.04	1.54	0.89–2.71	72.1%
Age	1.01	0.98–1.04	0.42	1.01	0.97–1.05	32.4%
Sex (Male)	1.12	0.71–1.77	0.61	1.09	0.68–1.82	28.7%
Stage (III/IV)	1.89	1.21–2.95	0.005	1.91	1.18–3.12	92.3%

Results from a multivariable Cox proportional hazards model assessing the impact of clinical factors and biomarker expression levels on survival outcomes. Hazard Ratios (HRs) for biomarkers compare the high-expression group (or the low-expression group for PPARG) to the reference group, adjusted for age, sex, and disease stage. The “Bootstrap Mean” and “95% Bootstrap CI” represent the mean HR and the 95% confidence interval derived from 1000 bootstrap resamples to assess the model’s stability. “% Significant Iterations” indicates the proportion of bootstrap samples in which the variable remained statistically significant (*p* < 0.05). Variables with high bootstrap significance (e.g., IL6, TNF, PPARG, Stage) demonstrate robust associations with survival, while AKT1 and STAT3 show less stability despite nominal significance in the original model.

**Table 9:** Summary of Protein Expression Validation.

Gene	Normal Expression (Score)	CCA Expression (Score)	Change	IHC Score
*IL6*	Low (1.2)	High (3.4)	↑ 2.2	8.7
*TNF*	Low (1.5)	Medium (2.8)	↑ 1.3	7.9
*PPARG*	Medium (2.8)	Low (1.6)	↓ 1.2	8.2
*AKT1*	Low (1.8)	High (3.6)	↑ 1.8	9.1
*STAT3*	Medium (2.4)	High (3.2)	↑ 0.8	7.5

Scores represent mean protein intensity on a 0–3 scale (0 = none, 1 = weak, 2 = moderate, 3 = strong). Normal = adjacent non-tumor tissue; CCA = cholangiocarcinoma tissue. Change = CCA − Normal intensity. IHC Score = composite score for CCA tissue only, calculated as (Intensity × % positive cells)/10 (range 0–10). This score provides a semi-quantitative measure of overall protein abundance by integrating staining intensity and the proportion of positive tumor cells. IL6—Interleukin 6, TNF—Tumor Necrosis Factor, AKT1—AKT Serine/Threonine Kinase 1, STAT3—Signal Transducer and Activator of Transcription 3, PPARG—Peroxisome Proliferator Activated Receptor Gamma.

## Data Availability

The data supporting the findings of this study are available in the article. Publicly available datasets were analyzed in this study, including the TCGA Cholangiocarcinoma (CHOL) cohort via the UCSC Xena Browser and Gene Expression Omnibus (GEO) Series GSE107943 (CCA validation), GSE23343 (Type 2 Diabetes liver), GSE58208 (HBV-infected liver), and GSE89632 (Metabolic dysfunction/steatosis). Additional resources included the Human Protein Atlas (v24.0) for protein expression data, the STRING database for PPI networks, and the KEGG Pathway, Reactome Pathway, and Gene Ontology databases for bioinformatics analyses. Data generated during this study are available from the corresponding author upon reasonable request.

## Abbreviations

**English Abbreviations**	**Full name in English**
T2D	Diabetes mellitus type 2
TCGA	The Cancer Genome Atlas
GEO	Gene Expression Omnibus
HPA	Human Protein Atlas
DEG	Differentially Expressed Gene
PPI	Protein–Protein Interaction
KEGG	Kyoto Encyclopedia of Genes and Genomes
GO	Gene Ontology
GSEA	Gene Set Enrichment Analysis
OS	Overall Survival
DFS	Disease-Free Survival
HR	Hazard Ratio
CI	Confidence Interval
FDR	False Discovery Rate
RNA-seq	RNA Sequencing
IHC	Immunohistochemistry
IL6	Interleukin 6
TNF	Tumor Necrosis Factor
PPARG	Peroxisome Proliferator-Activated Receptor Gamma
AKT1	AKT Serine/Threonine Kinase 1
STAT3	Signal Transducer and Activator of Transcription 3
NFKB1	Nuclear Factor Kappa B Subunit 1
VEGFA	Vascular Endothelial Growth Factor A
EGFR	Epidermal Growth Factor Receptor
KRAS	KRAS Proto-Oncogene, GTPase
TP53	Tumor Protein P53
MYC	MYC Proto-Oncogene, bHLH Transcription Factor
